# Increased cardiac macrophages in *Sorbs2*-deficient hearts: revealing a potential role for macrophage in responding to embryonic myocardial abnormalities

**DOI:** 10.3389/fgene.2024.1525931

**Published:** 2025-01-15

**Authors:** Beibei Hu, Xiangyang Liu, Shanshan Xiong, Qin Gong, Junjie Yang, Hongjun Shi, Min Zhang, Fei Liang, Zhen Zhang

**Affiliations:** ^1^ Pediatric Translational Medicine Institute and Pediatric Congenital Heart Disease Institute, Shanghai Children’s Medical Center, Shanghai Jiao Tong University School of Medicine, Shanghai, China; ^2^ School of Medicine, Westlake University, Hangzhou, Zhejiang, China; ^3^ Westlake Laboratory of Life Sciences and Biomedicine, Hangzhou, Zhejiang, China; ^4^ Westlake Institute for Advanced Study, Hangzhou, Zhejiang, China; ^5^ Shanghai United International School (Gubei Campus), Shanghai, China; ^6^ Neonatal Intensive Care Unit, Shanghai Children’s Medical Center, Shanghai Jiao Tong University School of Medicine, Shanghai, China; ^7^ Shanghai Collaborative Innovative Center of Intelligent Medical Device and Active Health, Shanghai University of Medicine and Health Sciences, Shanghai, China

**Keywords:** macrophage, Sorbs2, cardiac septal defect, valve formation, adaptive response

## Abstract

Macrophages are known to support cardiac development and homeostasis, contributing to tissue remodeling and repair in the adult heart. However, it remains unclear whether embryonic macrophages also respond to abnormalities in the developing heart. Previously, we reported that the structural protein Sorbs2 promotes the development of the second heart field, with its deficiency resulting in atrial septal defects (ASD). In analyzing RNA-seq data, we noted an upregulation of macrophage-related genes in *Sorbs2* ^−/−^ hearts. Immunostaining and lineage-tracing confirmed an increase in macrophage numbers, underscoring a macrophage response to myocardial abnormalities. Partial depletion of macrophages led to downregulation of genes involved in lipid metabolism, muscle development and organ regeneration, alongside upregulation of genes associated with DNA damage-induced senescence and cardiomyopathy. Additionally, a non-significant increase in septal defects in macrophage-depleted *Sorbs2* ^−/−^ hearts suggests a potential reparative function for macrophages in maintaining structural integrity. Valve formation, however, remained unaffected. Our findings suggest that embryonic macrophages might sense abnormalities in embryonic cardiomyocytes and could adaptively support cardiac structure and function development in response to myocardial abnormalities.

## Introduction

Cardiac morphogenesis initially involves the coordinated actions of progenitor cells, which give rise to diverse cell types within the heart and drive the initial stages of heart formation, establishing the basic structure and organization of the heart (Van Vliet et al., 2012). However, myocardial development is also essential for cardiac morphogenesis, providing the contractile force needed for circulation and shaping the developing heart (Taber et al., 2010). Cardiomyocyte differentiation initiates the expression of sarcomeric proteins such as actin and myosin. These proteins subsequently assemble into the complex and highly ordered sarcomere, the basic structural and functional unit of myofibrils. Over time, more accessory proteins are added into the rudimentary assemblies to form a mature muscle contractile apparatus (Guo and Pu, 2020). Mutations in major sarcomeric genes are commonly associated with cardiomyopathy but can also lead to abnormal non-syndromic congenital heart defects such as ASD (Yasuhara and Garg, 2021). Sorbs2 (sorbin and SH3 domain-containing 2) is an accessory protein located at the Z disk and intercalated disk in cardiomyocytes, crucial for sarcomere organization and the structural integrity of the intercalated disk (Ding et al., 2020; Wang et al., 1997). Knockout of *Sorbs2* causes arrhythmogenic and dilated cardiomyopathies (Ding et al., 2020; McLendon et al., 2020). *Sorbs2* deficiency also leads to incomplete penetrance of ASD (Liang et al., 2021).

Beyond the intrinsic structural components within cardiomyocytes, other cell types in the myocardial microenvironment, such as fibroblasts and immune cells, contribute to cardiac morphogenesis and maintenance (Ding et al., 2022). In the embryonic heart, macrophages initially present in the subepicardial space later spread to deeper layers (Gula et al., 2021), including the bulbar and atrioventricular cushions (Shigeta et al., 2019). During mammalian heart development, macrophages participate in coronary vessel development, lymphangiogenesis, and cardiac valve shaping (Cahill et al., 2021; Leid et al., 2016; Shigeta et al., 2019). In mature hearts, cardiac macrophages contribute to electrical conduction, maintain homeostasis, and respond to pathological conditions to affect post-injury repair and remodeling (Moskalik et al., 2022). However, the macrophage response to pathological conditions in the embryonic heart remains unclear.

We previously reported that *Sorbs2* is essential for atrial septum development, with *Sorbs2* knockout causing ASD in about 40% of embryos (Liang et al., 2021). Interestingly, RNA-seq data from E10.5 *Sorbs2* ^−/−^ embryos revealed upregulated macrophage gene expression. To determine whether this upregulation is due to increased macrophages in the heart, we used immunofluorescent staining and macrophage lineage-tracing to evaluate macrophage number and distribution in E12.5 hearts. Results showed an increase in macrophages within embryonic hearts. Partial ablation of cardiac resident macrophages significantly altered the cardiac transcriptome at E12.5. Although we did not observe valve malformation, there was a non-significant increase in septal defect penetrance. Collectively, our results indicate that cardiac macrophages respond to structural gene mutations and might play a reparative role in myocardial morphogenesis and function.

## Results

### Increased expression of macrophage-related genes in *Sorbs2* ^−/−^ hearts

**FIGURE 1:**
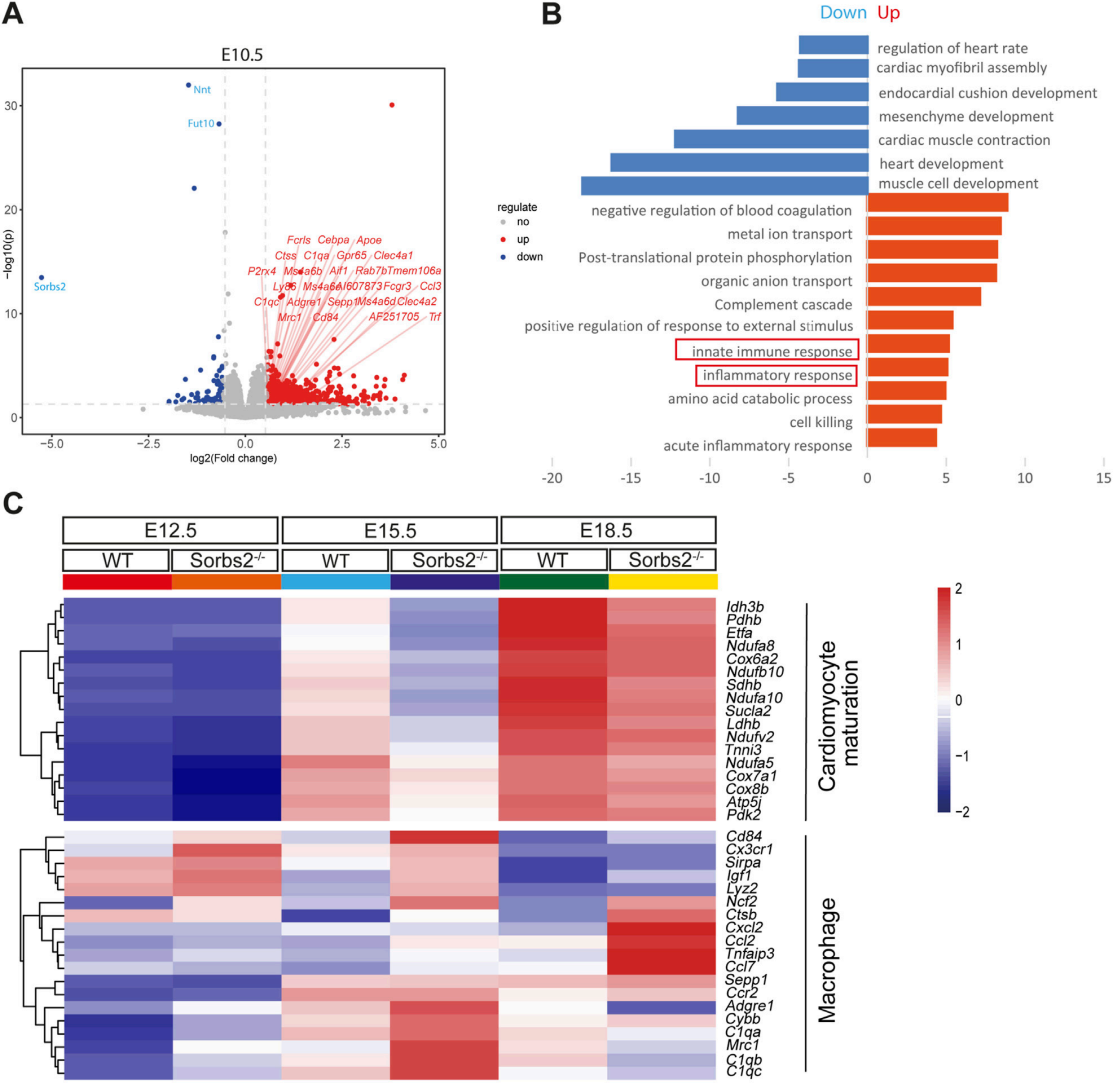
Increased expression of macrophage-related genes in *Sorbs2* ^−/−^ embryonic heart **(A)** Volcano plot illustrating differentially expressed genes (DEGs) at embryonic day 10.5. Each point corresponds to an individual gene. Genes with statistically significant differential expression (*p* < 0.05 and an absolute log_2_ (fold change) > 0.58) are highlighted. Red, upregulated genes. Blue, downregulated genes. Gray, non-significant genes. Macrophage-related genes are marked with red slashes. **(B)** Enrichment analysis of DEGs in E12.5 embryonic ventricles (homozygous vs. wild type). **(C)** Heatmap visualizing gene expression in embryonic ventricles at E12.5, E15.5, and E18.5. Color tints indicate expression levels. Genes in the upper section are involved in cardiomyocyte maturation, while those in the lower section are linked to macrophages.

In analyzing the transcriptomic data of E10.5 embryos (Liang et al., 2021), we noted upregulation of macrophage-related genes, such as *C1q1*, *Adgre1*, and *Mrc1*, in *Sorbs2* ^−/−^ mutants (Figure 1A). However, *Sorbs2* is not expressed in macrophages but is highly expressed in embryonic hearts (Supplementary Figure S1). We hypothesized the upregulation of macrophage-related genes occurs within the heart. Since macrophages start to populate hearts as early as E9.5 (Epelman et al., 2014), we collected E12.5, E15.5 and E18.5 ventricles to perform RNA-seq (Supplementary Tables S1–3). We selected genes significantly downregulated in E12.5 mutant hearts [log_2_ (fold change) <-0.58, *p* < 0.05] to perform GO analysis and found these genes enriched in pathways related to the electron transport chain and mitochondrial translation elongation (Figures 1B, C), suggesting that *Sorbs2* positively regulates myocardial maturation. Using the same threshold, we selected genes significantly upregulated in E12.5 mutant hearts to perform GO analysis. Interestingly, these genes are enriched in pathways regulating immune response (Figure 1B). Upon closer examination, we observed differential expression of macrophage marker genes such as *Cx3cr1* and *Lyz2* (Figure 1C)*,* suggesting that macrophages may be activated in *Sorbs2* ^−/−^ hearts. These changes in gene expression patterns persisted throughout E18.5 (Figure 1C). Taken together, our RNA-seq results revealed that *Sorbs2* deficiency impairs myocardial maturation and triggers a response in macrophages.

### Increased number of macrophages in *Sorbs2* ^−/−^ hearts

**FIGURE 2:**
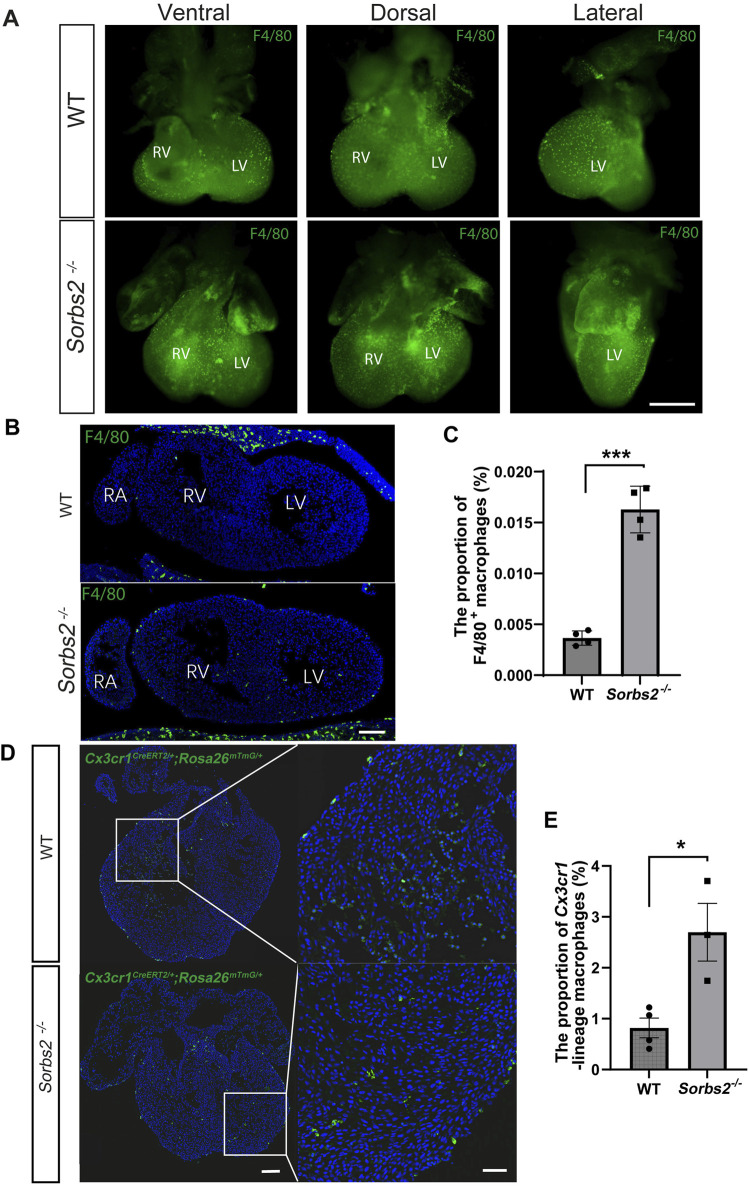
Increased number of macrophages in embryonic *Sorbs2* ^−/−^ hearts **(A)** Whole-mount immunostaining of E12.5 embryonic hearts. Samples were stained with anti-F4/80 antibody (green) to visualize macrophages. RV, right ventricle. LV, left ventricle. Scale bar, 500 μm. **(B)** Representative images of E12.5 heart sections immunostained with anti-F4/80 antibody (green) and DAPI (blue). RV, right ventricle. LV, left ventricle. RA, right atrium. Scale bar, 100 μm. **(C)** Quantification of F4/80^+^ macrophages (n = 4 per group). *, *p* < 0.001. Nested ANOVA test. **(D)** Representative images of *Cx3cr1*-lineage macrophages (green) in E12.5 hearts. Scale bar, 100 μm for the low magnification and 50 μm for the high magnification. **(E)** Quantification of *Cx3cr1*-lineage macrophages (n = 4 for WT group, n = 3 for *Sorbs2* ^−/−^ group). *, *p* < 0.05. Nested ANOVA test.

The increased expression of macrophage marker genes in *Sorbs2* ^−/−^ embryonic hearts prompted us to examine macrophage numbers in mutant hearts. To this end, we performed whole-mount immunostaining on E12.5 hearts, using an antibody against the pan macrophage marker F4/80. Indeed, it revealed increased macrophages in the ventricles of E12.5 *Sorbs2* ^−/−^ hearts (Figure 2A). In section analysis, macrophages were mainly distributed under the epicardium and in the outer layer of the myocardium (Figure 2B). Consistently, sections of *Sorbs2* ^−/−^ heart displayed increased macrophage counts (Figures 2B, C).

Next, we used macrophage lineage-tracing to further validate this observation. *Cx3cr1* is a marker of embryonic heart macrophage (Leid et al., 2016). We bred *Cx3cr1* ^*CreERT2*^ and *Rosa26* ^*mTmG*^ alleles into *Sorbs2* ^−/−^ mice. Tamoxifen-induced CreERT-mediated recombination in the *Rosa26* locus led to EGFP expression in *Cx3cr1*-lineage macrophages, confirming increased macrophages in the ventricular walls of E12.5 *Sorbs2* ^−/−^ hearts (Figures 2D, E). These data indicate that increased expression of macrophage-related genes results from the increased number of macrophages in *Sorbs2* ^−/−^ hearts.

### Macrophage-specific CreERT-induced DTA significantly reduced cardiac macrophages

Macrophages are vital residents of the developing heart. During heart development, tissue resident macrophages regulate coronary vessel formation and lymphatic network development (Cahill et al., 2021; Leid et al., 2016). They are also essential for the developmental remodeling of cardiac valves (Shigeta et al., 2019). The increased macrophages in *Sorbs2* ^−/−^ hearts led us to question whether macrophages might play an unknown role in the abnormal embryonic hearts.

**FIGURE 3:**
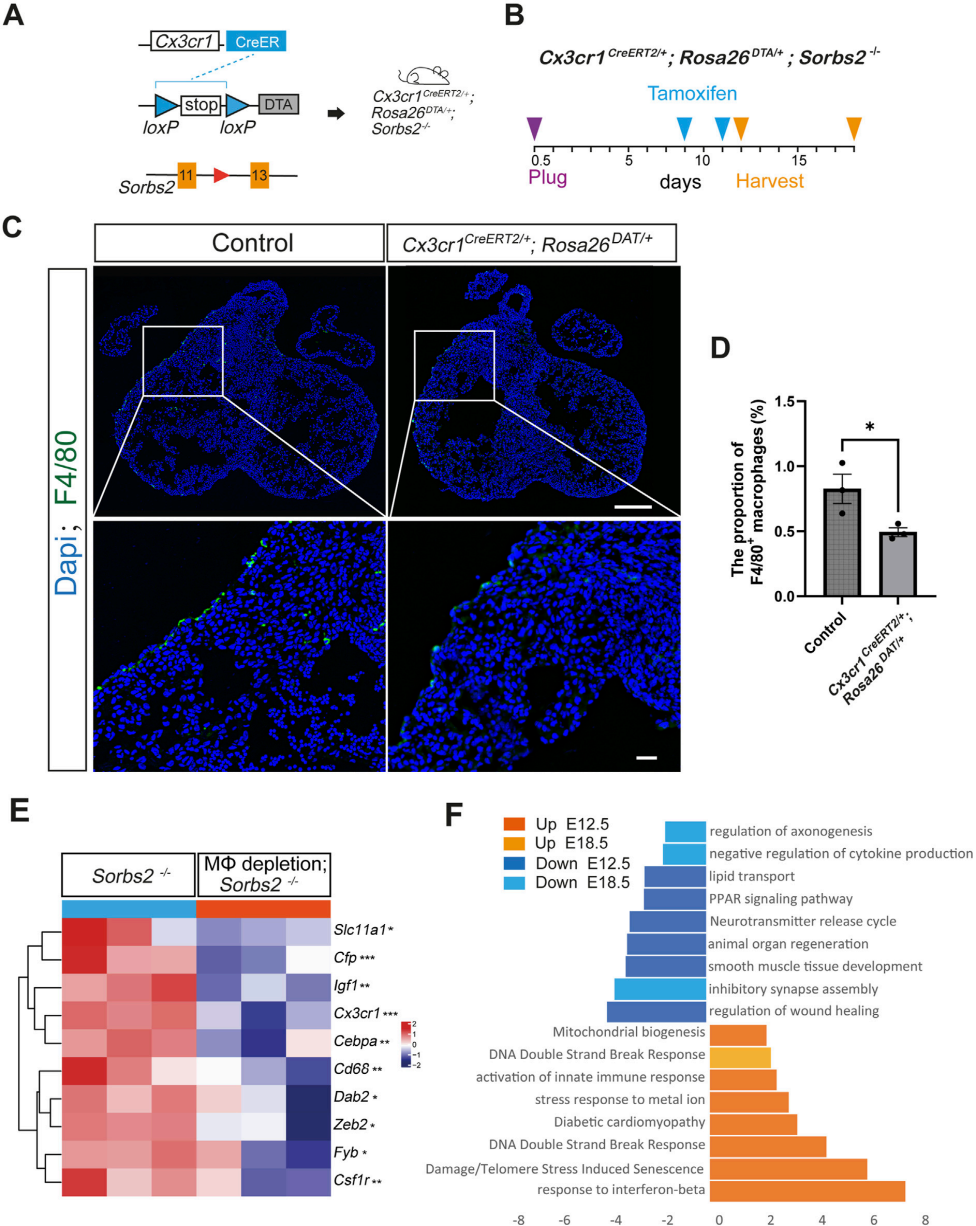
Transcriptomic changes in macrophage-depleted *Sorbs2* ^−/−^ hearts **(A)** Strategies for macrophage depletion in *Sorbs2* ^−/−^ mice. **(B)** Experimental design. Tamoxifen was administered at E9.5 and E11.5 via oral gavage. Hearts were harvested at E12.5 or E18.5. **(C)** Representative immunofluorescent images of E12.5 hearts stained with anti-F4/80 antibody (green) and DAPI (blue). Scale bar, 200 μm for the low magnification and 400 μm for the high magnification. **(D)** Quantification of F4/80^+^ macrophages (n = 3 per group). *, *p* < 0.05. Nested ANOVA test. **(E)** Heatmap illustrating expression levels of macrophage-related gene in E12.5 heart ventricles. *, *p* < 0.05. **, *p* < 0.01. ***, *p* < 0.001. MΦ, macrophage. **(F)** Enrichment analysis of DEGs in embryonic ventricles at E12.5 and E18.5 (*Cx3cr1* ^*CreERT2/+*^; *Rosa26* ^*DTA/+*^; *Sorbs2* ^−/−^ vs. *Sorbs2* ^−/−^).

To this end, we took a cell depletion approach with the *Rosa26* ^*DTA*^ alleles, which encodes cytotoxic diphtheria toxin A (DTA) after Cre-induced recombination removes the STOP element, therefore killing cells that express DTA (Ivanova et al., 2005). Tamoxifen was administered to *Cx3cr1* ^*CreERT2/+*^; *Rosa26* ^*DTA/+*^ mice at E9.5 and E11.5 through oral gavage, and hearts were collected at E12.5 for F4/80 immunostaining to check the efficiency of macrophage depletion (Figures 3A, B). Results showed that macrophages were decreased in *Cx3cr1* ^*CreERT2/+*^; *Rosa26* ^*DTA/+*^ hearts (Figure 3C). The quantification indicated that macrophage numbers significantly decreased and the reduction ratio was about 40% (Figure 3D).

### Transcriptomic changes induced by macrophage depletion

We obtained *Cx3cr1* ^*CreERT2/+*^; *Rosa26* ^*DTA/+*^ *;Sorbs2* ^−/−^ mice to examine the effect of macrophage depletion on *Sorbs2* ^−/−^ hearts. We collected E12.5 ventricles for RNA-seq (Supplementary Table S4). Results showed that macrophage marker genes, such as *Cx3cr1*, *Cd68* and *Csf1r*, were significantly reduced compared with control littermate embryos (Figure 3E), verifying reduced macrophages. Pathway analysis showed downregulated genes involved in organ regeneration, lipid metabolism, muscle development, and neurotransmitter signaling, while upregulated genes were associated with innate immune response activation, DNA damage-induced senescence, diabetic cardiomyopathy, and mitochondrial biogenesis (Figure 3F). These results suggest that macrophage depletion impairs cardiac metabolism and causes cardiomyocyte damage in mid-gestation stage. By E18.5, transcriptomic changes between macrophage-depleted and non-depleted groups were minimal (Supplementary Table S5), indicating a transient effect of our macrophage depletion strategy, though we noted continued downregulation in neural development genes and upregulation in DNA damage response genes (Figure 3F).

### Partial macrophage depletion slightly increased the penetrance of structural cardiac defects in *Sorbs2* ^−/−^ hearts

**FIGURE 4:**
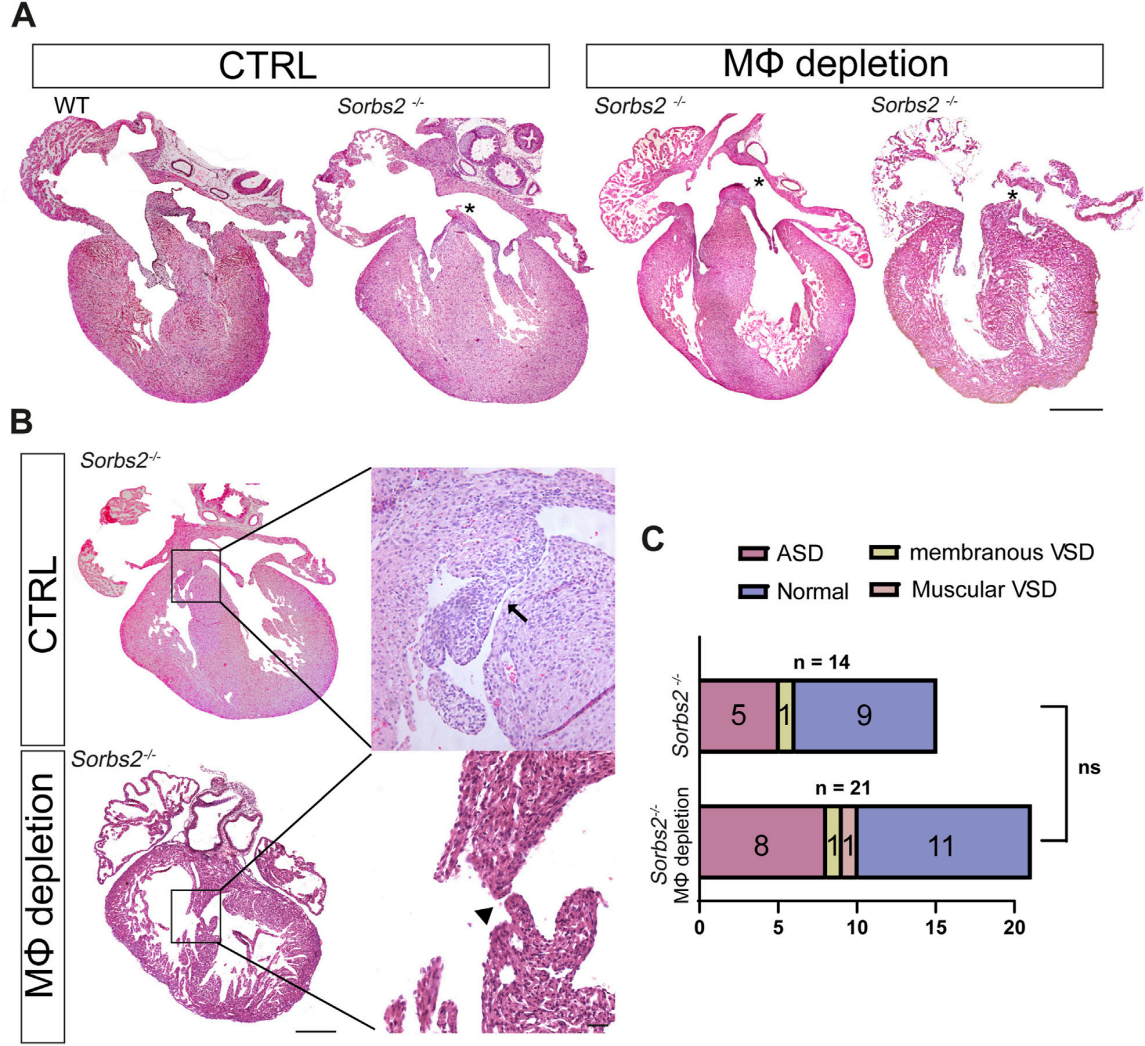
Septal defects in macrophage-depleted and non-depleted *Sorbs2* ^−/−^ hearts **(A)** Hematoxylin and eosin (HE)-stained paraffin sections of E18.5 hearts. Asterisk indicates ASD. Scale bar, 200 μm. **(B)** HE-stained paraffin sections of E18.5 hearts. Boxed areas are magnified to highlight VSDs. Arrow, membranous VSD. Arrowhead, muscular VSD. Scale bar, 200 μm for the low magnification and 500 for the high magnification. **(C)** Penetrance of ASD and VSD in macrophage-depleted and non-depleted *Sorbs2* ^−/−^ hearts. Ns, non-significant. Chi-square test.

As previously reported, about 40%–60% *Sorbs2* ^−/−^ mice died within 1 week after birth, with about 40% presenting atrial septal defect (ASD) (Zhang et al., 2016; Liang et al., 2021). We wondered whether the macrophage increase in *Sorbs2^−/−^* hearts is an attempt to repair the structural defect caused by *Sorbs2* deficiency. To this end, we collected embryos at E18.5 to check cardiac structure defects. Genotype distribution ratios were consistent with Mendel’s law, suggesting no embryo loss during embryonic development (Supplementary Table S6). In Tamoxifen-administered hearts, all 8 macrophage-depleted WT and *Sorbs2* ^−/−^ hearts exhibited no structural abnormalities, whereas we observed ASD in *Sorbs2* ^−/−^ hearts as reported previously (Figure 4A). None of *Sorbs2* ^*−/−*^ hearts showed conotruncal defects, but we noted membranous ventricular septal defect (VSD) in both macrophage-depleted and non-depleted *Sorbs2* ^−/−^ hearts (Figures 4B, C). Among 21 macrophage-depleted *Sorbs2* ^*−/−*^ embryos, 8 showed ASD, 1 showed membranous VSD, and 1 showed muscular VSD (Figures 4B,C). In contrast, among the 14 macrophage-non-depleted *Sorbs2* ^*−/−*^ embryos, 5 showed ASD and 1 showed membranous VSD, with one embryo exhibiting both ASD and VSD (Figure 4C). Compared to macrophage-non-depleted *Sorbs2* ^*−/−*^ embryos, there was a trend toward increased penetrance of cardiac defects in macrophage-depleted *Sorbs2* ^*−/−*^ embryos, though the difference was not significant (Figure 4C). This non-significant increase in cardiac defect penetrance, particularly the occurrence of one muscular VSD in macrophage-depleted *Sorbs2* ^*−/−*^ embryos, suggests that macrophages might play a repairing role in cardiac development.

### Ablation of cardiac resident macrophages did not affect valve development

**FIGURE 5:**
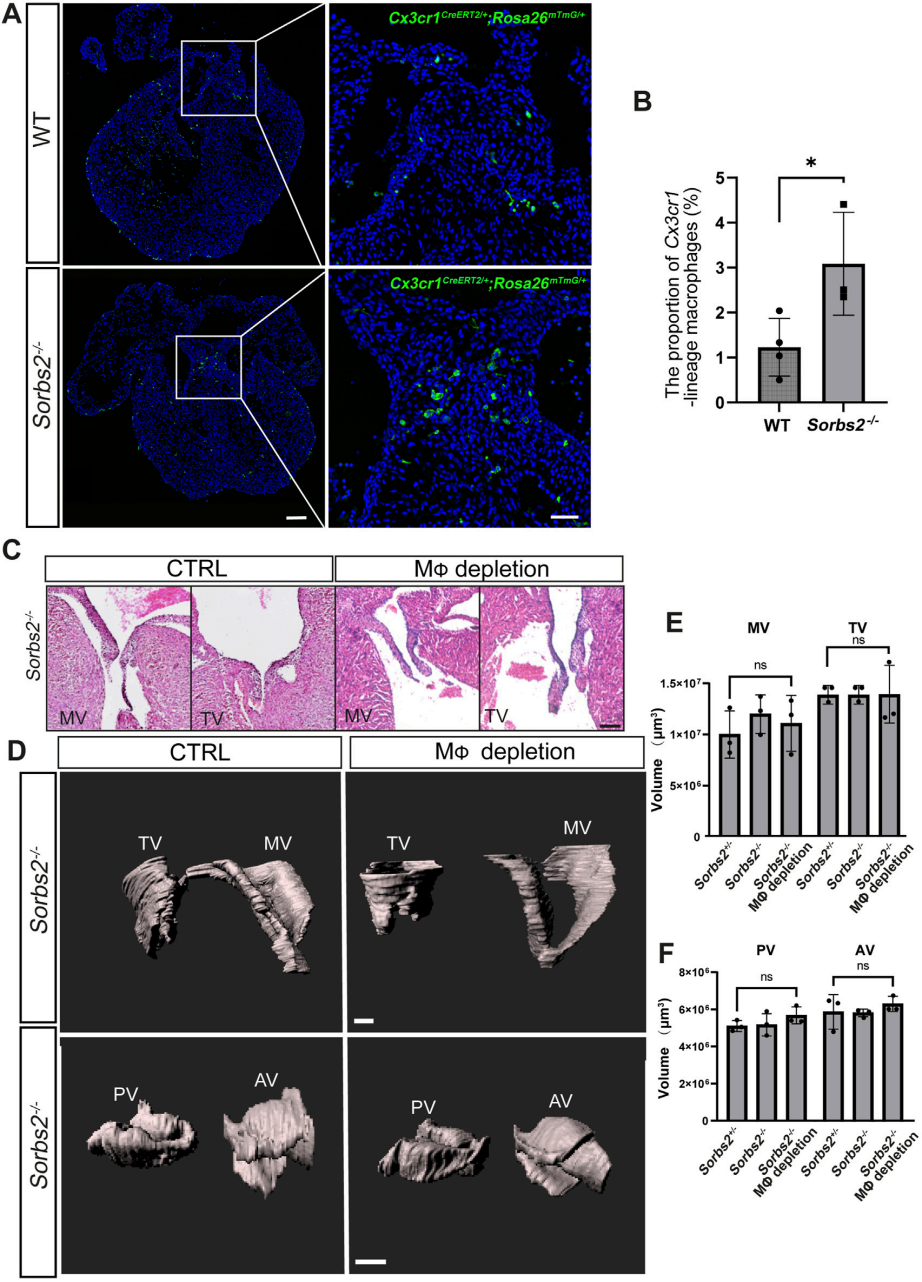
Morphological analysis of valves in macrophage-depleted and non-depleted *Sorbs2* ^−/−^ hearts **(A)** Representative images of *Cx3cr1*-lineage macrophages (green) in the endocardial cushion of E12.5 hearts at E12.5d. Scale bar, 100 μm for low magnification and 500 μm for high magnification. **(B)** Quantification of *Cx3cr1*-lineage macrophages in the endocardial cushion (n = 4 for the WT group, n = 3 for the *Sorbs2* ^−/−^ group). *, *p* < 0.05. Nested ANOVA test. **(C)** HE-stained paraffin sections of E18.5 hearts showing the mitral and tricuspid valves. MV, mitral valve. TV, tricuspid valve. Scale bar, 50 μm. **(D)** Representative 3D reconstructions of E18.5 mitral, tricuspid, pulmonary, and aortic valves through manual surface rendering in Imaris. MV, mitral valve. TV, tricuspid valve. PV, pulmonary valve. AV, aortic valve. Scale bar, 100 μm for the top row, 150 μm for the bottom row. **(E)** Quantification of the mitral and tricuspid valve volume (n = 3 per group). Ns, non-significant. One-way ANOVA test. MV, mitral valve. TV, tricuspid valve. **(F)** Quantification of the pulmonary and aortic valve volume (n = 3 per group). Ns, non-significant. One-way ANOVA test.

A previous report shows that cardiac resident macrophages are required for valve formation (Shigeta et al., 2019). We also noted an increase in the number of *Cx3cr1*-lineage macrophages in the endocardial cushions of E12.5 hearts (Figures 5A, B). Therefore, we harvested E18.5 embryos to perform a morphological analysis of valves. In histological sections, we did not observe any obvious morphological abnormality in mitral and tricuspid valves of both macrophage-depleted and non-depleted *Sorbs2* ^*−/−*^ hearts (Figure 5C). To obtain a whole view of cardiac valves, we used light sheet fluorescence microscopy to reconstruct a three-dimensional visualization of valves. Surface rendering was applied to delineate and calculate the volumes of the mitral, tricuspid, pulmonary and aortic valves. We did not detect any obvious abnormality in cardiac valves (Figure 5D). Quantification of valve volume showed no significant differences in any of cardiac valves between macrophage-depleted and non-depleted *Sorbs2* ^*−/−*^ groups, and nor in comparisons between *Sorbs2* ^*+/−*^ and *Sorbs2* ^*−/−*^ groups (Figures 5E, F).

## Discussion

Our study sheds light on a previous unknown role of macrophages in the embryonic heart under conditions of structural gene mutation, specifically the Sorbs2 knockout model. We observed increased macrophage numbers in the embryonic hearts of *Sorbs2* ^*−/−*^ mice. This response likely represents an adaptive reaction to structural abnormalities in the myocardium, suggesting that macrophages may have a supportive role in cardiac morphogenesis under compromised conditions.

Through macrophage depletion, we observed a profound transcriptomic shifts indicating impaired metabolic and developmental pathways in E12.5 *Sorbs2* ^*−/−*^ hearts. Downregulation of genes related to lipid metabolism, animal organ regeneration, and muscle development in macrophage-depleted hearts suggests that macrophages contribute to the metabolic support required for myocardial maturation. In mice, the completion of the placenta formation occurs at E12.5 and subsequently and the partial pressure of oxygen in the fetal circulation increases (Hemberger et al., 2020; Slaats et al., 2020). Meanwhile the heart muscle experiences substantial thickening and mitochondrial morphology and function, crucial for substrate oxidative phosphorylation, undergo a process of maturation at this time (Barak et al., 2019; Porter et al., 2011). On the molecular level, heart glucose uptake decreases after E12 and the expression of glycolytic enzymes, including *Glut1*, *Pdk1*, and *Ldha*, becomes decreased along ventricular myocyte thickening during mid-to late-gestational stages (Menendez-Montes et al., 2016; Nakano et al., 2017). Our data indicate that increased macrophages attenuate the impacts on lipid metabolism and muscle development induced by *Sorbs2* deficiency. Additionally, the observed increase in DNA damage response genes and cardiomyopathy hints at increased cellular stress in macrophage-depleted hearts, further highlighting their protective role in managing cellular stress and maintaining cardiac integrity. Therefore, macrophages might be safeguards for the metabolic shift and myocardial growth at this stage.

While macrophage depletion did not significantly increase the penetrance of septal defects, the observed trend still implies that macrophages might play a reparative role in the presence of structural cardiac abnormalities. The appearance of VSD in macrophage-depleted *Sorbs2* ^*−/−*^ hearts, which were not seen in non-depleted *Sorbs2* ^*−/−*^ hearts, supports the idea that macrophages may help mitigate certain developmental defects in compromised embryonic hearts. These findings point to a potential role for cardiac macrophages as adaptive responders to structural gene mutations. Given the established role of macrophages in cardiac regeneration and repair (de Couto, 2019), our primitive findings could extend this role to include compensatory repair during morphogenesis.

In contrast, macrophage depletion did not significantly impact valve formation or volume, despite prior reports indicating their role in valve development (Shigeta et al., 2019). This discrepancy could be due to the partial and/or transient nature of macrophage depletion in our study. New depletion strategies that achieve complete and consistent macrophage removal could help resolve this inconsistency. Although partial macrophage depletion has provided initial insights into a possible compensatory role of macrophages during embryonic myocardial development, the incomplete depletion may have limited our ability to observe the full impact of macrophage absence on heart development. Further studies with more targeted macrophage ablation techniques, or using models that allow for more complete and temporally controlled macrophage depletion, would clarify these effects in responding to myocardial abnormality and contributing to different aspects of cardiac morphogenesis.

A significant limitation of our study is the incomplete understanding of the mechanisms underlying the observed increase in macrophages in *Sorbs2* ^*−/−*^ hearts. Although our findings indicate an upregulation of macrophage-related genes and an increase in macrophage numbers, we have not addressed that it is due to increased macrophage recruitment or proliferation. It remains unclear whether the increase in macrophages results directly from changes within cardiomyocytes due to *Sorbs2* deficiency or from secondary signals generated by other cells or altered extracellular matrix components in the myocardial environment. Identifying the sources and nature of these signals would provide valuable insights into how structural gene mutations influence immune cell behavior.

In conclusion, our findings highlight the adaptive role of cardiac macrophages in response to structural gene mutations. While macrophages are known to be vital for normal heart development, our study provides evidence that they may also mitigate developmental defects in structurally compromised hearts. Future work should investigate the specific signaling pathways that mediate macrophage responses to myocardial abnormalities, as well as potential therapeutic strategies for modulating macrophage activity to support heart development in congenital heart disease.

## Methods

### Mice

The mouse strains utilized in this study comprise *Sorbs2* ^-^ (Liang et al., 2021), *Cx3cr1* ^*CreERT2*^ (Xu et al., 2020), *Rosa26* ^*DTA*^ (Ivanova et al., 2005) and *Rosa26* ^*mTmG*^ (Muzumdar et al., 2007). The *Cx3cr1* ^*CreERT2*^ allele was a gift from Dr. Bo Peng’s lab (Fudan University, Shanghai, China). All strains were backcrossed with C57BL/6 to ensure the consistent genetic background. Tamoxifen was administered at E9.5 and E11.5 through oral gavage. Mice were maintained under specific pathogen-free conditions in the animal facility at Shanghai Children’s Medical Center. All animal procedures adhered to the guidelines set by the Institutional Animal Care and Use Committee of the Shanghai Children’s Medical Center, affiliated with the Shanghai Jiao Tong University School of Medicine.

### Histological analyses

For the preparation of frozen sections, dissected embryonic hearts were fixed in 4% paraformaldehyde for 20 min at 4°C, followed by equilibration in a 30% sucrose PBS solution overnight at 4°C.Subsequently, the hearts were embedded in 100% OCT compound within Cryomolds. The prepared blocks were immediately frozen at −80°C. The sample blocks were sectioned into 10 μm thin slices using a Leica CM3050S cryostat. For paraffin sections, the dissected embryonic hearts were fixed in 4% paraformaldehyde for 24 h at 4°C. The fixed hearts were rinsed with PBS, then subjected to a graded ethanol series (30%, 50%, 70%, 80%, 95%, 100%) for complete dehydration. Typically, each ethanol step was maintained for 30 min, followed by the embedding process. Upon completion of dehydration, the hearts were soaked in xylene for 30 min, followed by overnight paraffin infiltration. Finally, the hearts were processed for embedding in paraffin. The sample blocks were sectioned into 5 μm thin slices.

The sections were dewaxed with xylene and subsequently rinsed with ethanol. The sections were stained with a hematoxylin dye solution for a duration varying between 5 and 20 min, followed by a rinse with running water. The sections were then subjected to a 30-second differentiation process using a differentiation solution and subsequently rinsed with running water for 5 min. The slides were then immersed in eosin dye for 2 min. This was followed by conventional procedures for dehydration, clearing, and mounting.

### Immunofluorescent staining

The frozen sections were permeabilized in 0.5% Triton X-100/phosphate-buffered saline (PBS) for 20 min, followed by blocking in 3% bovine serum albumin/PBS for 1 h. The sections were then stained with F4/80 antibodies (1:400, ab16288; Abcam). The nuclei were subsequently stained with 4′,6-diamidino-2-phenylindole (DAPI). Fluorescent images were captured using a high-resolution fluorescence microscope.

### Whole-mount immunostaining

Initially, the samples are fixed in 4% paraformaldehyde/phosphate-buffered saline (PFA/PBS), followed by a sequential dehydration and rehydration process. Subsequently, the samples are treated with proteinase K and then inactivated with hydrogen peroxide (H_2_O_2_). Thereafter, blocking is performed, followed by the incubation with primary and secondary antibodies, and subsequent multiple washes. Afterwards, the Elite ABC reagent is prepared and incubated. Following staining with DAB reagent, the samples are transferred to PBS, fixed again in 4% PFA, and finally dehydrated and stored in 100% methanol.

### RNA-Seq

Total RNA of E12.5, E15.5 and E18.5 cardiac ventricles were isolated using TRizol reagent (Thermo Fisher Scientific; 15596018). Library preparation and transcriptome sequencing on an Illumina HiSeq platform were performed by Novogene Bioinformatics Technology Co., Ltd. to generate 100-bp paired-end reads. HTSeq v0.6.0 was used to count the read numbers mapped to each gene, and fragments per kilobase of transcript per million fragments mapped (FPKM) of each gene were calculated. We used FastQC to control the quality of transcriptome sequencing data. The expression level of each gene under different treatment conditions was obtained by HTSeq-count after standardization. The differentially expressed genes were analyzed by DESeq2 package (version 1.42.0). Functional enrichment of differentially expressed genes was analyzed on Metascape website. Heatmaps were created by the Pheatmap package (version 1.0.12) in R (version 4.3.1). scRNA-seq data for embryonic hearts (GSE150817) were retrieved from the Gene Expression Omnibus (GEO) database. Seurat toolkit (version 4.3.0) was used for scRNA-seq analysis. After data integration, batch effect elimination, normalization, and scaling, different cell populations were identified based on existing references. Gene expression was plotted using normalized read counts.

### Three-dimensional visualization of embryonic heart valves

For three-dimensional visualization of the embryonic heart valves, E18.5 embryos were harvested in PBS, fixed overnight in 10% formaldehyde and 2.5% glutaraldehyde, rinsed twice in PBS, and dehydrated through a graded series of alcohol (50%, 75%, 90%, and 100% twice) for 30 min per step at room temperature. The hearts were then transferred into specially designed glass tubes containing 100 µL of BABB solution (1:2 benzyl alcohol: benzyl benzoate) for complete clearing. A custom-developed device was used to mount the heart within the Zeiss Lightsheet Z.1 microscope chamber filled with 87% glycerol (RI = 1.45). 3D images were captured using the 561 nm laser line and detection optics 5x/0.16 (n = 1.45). The reconstruction of the image stacks was analyzed with Imaris 10.0 software. Surface rendering was applied to delineate and calculate the volumes of the mitral and tricuspid valves.

### Statistical analysis

Statistical significance was performed using a two-tailed Student’s t test, or nested ANOVA test as appropriate. Statistical significance is indicated by *, where *p* < 0.05, **, where *p* < 0.01, and ***, where *p* < 0.001.

## Data Availability

The original contributions presented in the study are publicly available. This data can be found here: RNA-seq data have been deposited in the NCBI’s Gene Expression Omnibus under accession GSE284404.

## References

[B1] Barak Y. Hemberger M. Sucov H. M. (2019). Phases and mechanisms of embryonic cardiomyocyte proliferation and ventricular wall morphogenesis. Pediatr. Cardiol. 40, 1359–1366. 10.1007/s00246-019-02164-6 31342113 PMC6786952

[B2] Cahill T. J. Sun X. Ravaud C. Villa Del Campo C. Klaourakis K. Lupu I.-E. (2021). Tissue-resident macrophages regulate lymphatic vessel growth and patterning in the developing heart. Development 148, dev194563. 10.1242/dev.194563 33462113 PMC7875498

[B3] De Couto G. (2019). Macrophages in cardiac repair: environmental cues and therapeutic strategies. Exp. Mol. Med. 51, 1–10. 10.1038/s12276-019-0269-4 PMC6923399 31857583

[B4] Ding S. Zhang X. Qiu H. Wo J. Zhang F. Na J. (2022). Non-cardiomyocytes in the heart in embryo development, health, and disease, a single-cell perspective. Front. Cell Dev. Biol. 10, 873264. 10.3389/fcell.2022.873264 36393852 PMC9661523

[B5] Ding Y. Yang J. Chen P. Lu T. Jiao K. Tester D. J. (2020). Knockout of SORBS2 protein disrupts the structural integrity of intercalated disc and manifests features of arrhythmogenic cardiomyopathy. J. Am. Heart Assoc. 9, e017055. 10.1161/JAHA.119.017055 32808564 PMC7660791

[B6] Epelman S. Lavine K. J. Beaudin A. E. Sojka D. K. Carrero J. A. Calderon B. (2014). Embryonic and adult-derived resident cardiac macrophages are maintained through distinct mechanisms at steady state and during inflammation. Immunity 40, 91–104. 10.1016/j.immuni.2013.11.019 24439267 PMC3923301

[B7] Gula G. Ruminski S. Niderla-Bielinska J. Jasinska A. Kiernozek E. Jankowska-Steifer E. (2021). Potential functions of embryonic cardiac macrophages in angiogenesis, lymphangiogenesis and extracellular matrix remodeling. Histochem Cell Biol. 155, 117–132. 10.1007/s00418-020-01934-1 33130914 PMC7847984

[B8] Guo Y. Pu W. T. (2020). Cardiomyocyte maturation: new phase in development. Circ. Res. 126, 1086–1106. 10.1161/CIRCRESAHA.119.315862 32271675 PMC7199445

[B9] Hemberger M. Hanna C. W. Dean W. (2020). Mechanisms of early placental development in mouse and humans. Nat. Rev. Genet. 21, 27–43. 10.1038/s41576-019-0169-4 31534202

[B10] Ivanova A. Signore M. Caro N. Greene N. D. Copp A. J. Martinez-Barbera J. P. (2005). *In vivo* genetic ablation by Cre-mediated expression of diphtheria toxin fragment A. Genesis 43, 129–135. 10.1002/gene.20162 16267821 PMC2233880

[B11] Leid J. Carrelha J. Boukarabila H. Epelman S. Jacobsen S. E. W. Lavine K. J. (2016). Primitive embryonic macrophages are required for coronary development and maturation. Circulation Res. 118, 1498–1511. 10.1161/CIRCRESAHA.115.308270 27009605 PMC5567774

[B12] Liang F. Wang B. Geng J. You G. Fa J. Zhang M. (2021). SORBS2 is a genetic factor contributing to cardiac malformation of 4q deletion syndrome patients. eLife 10, e67481. 10.7554/eLife.67481 34099102 PMC8186900

[B13] Mclendon J. M. Zhang X. Matasic D. S. London B. Boudreau R. L. (2020). Loss of SORBS2 in cardiomyocytes leads to dilated left ventricle cardiomyopathy in mice. FASEB J. 34, 1. 10.1096/fasebj.2020.34.s1.07385 PMC9333371 35730644

[B14] Menendez-Montes I. Escobar B. Palacios B. Gomez M. J. Izquierdo-Garcia J. L. Flores L. (2016). Myocardial VHL-HIF signaling controls an embryonic metabolic switch essential for cardiac maturation. Dev. Cell 39, 724–739. 10.1016/j.devcel.2016.11.012 27997827

[B15] Moskalik A. Niderla-Bielinska J. Ratajska A. (2022). Multiple roles of cardiac macrophages in heart homeostasis and failure. Heart Fail Rev. 27, 1413–1430. 10.1007/s10741-021-10156-z 34387811 PMC9197805

[B16] Muzumdar M. D. Tasic B. Miyamichi K. Li L. Luo L. (2007). A global double-fluorescent Cre reporter mouse. Genesis 45, 593–605. 10.1002/dvg.20335 17868096

[B17] Nakano H. Minami I. Braas D. Pappoe H. Wu X. Sagadevan A. (2017). Glucose inhibits cardiac muscle maturation through nucleotide biosynthesis. Elife 6, e29330. 10.7554/eLife.29330 29231167 PMC5726851

[B18] Porter G. A. JR. Hom J. Hoffman D. Quintanilla R. De Mesy Bentley K. Sheu S. S. (2011). Bioenergetics, mitochondria, and cardiac myocyte differentiation. Prog. Pediatr. Cardiol. 31, 75–81. 10.1016/j.ppedcard.2011.02.002 21603067 PMC3096664

[B19] Shigeta A. Huang V. Zuo J. Besada R. Nakashima Y. Lu Y. (2019). Endocardially derived macrophages are essential for valvular remodeling. Dev. Cell 48, 617–630. 10.1016/j.devcel.2019.01.021 30799229 PMC6440481

[B20] Slaats R. H. Schwach V. Passier R. (2020). Metabolic environment *in vivo* as a blueprint for differentiation and maturation of human stem cell-derived cardiomyocytes. Biochim. Biophys. Acta Mol. Basis Dis. 1866, 165881. 10.1016/j.bbadis.2020.165881 32562698

[B21] Taber L. A. Voronov D. A. Ramasubramanian A. (2010). The role of mechanical forces in the torsional component of cardiac looping. Ann. N. Y. Acad. Sci. 1188, 103–110. 10.1111/j.1749-6632.2009.05089.x 20201892 PMC2837544

[B22] Van Vliet P. Wu S. M. Zaffran S. Puceat M. (2012). Early cardiac development: a view from stem cells to embryos. Cardiovasc Res. 96, 352–362. 10.1093/cvr/cvs270 22893679 PMC3500045

[B23] Wang B. Golemis E. A. Kruh G. D. (1997). ArgBP2, a multiple Src homology 3 domain-containing, Arg/Abl-interacting protein, is phosphorylated in v-Abl-transformed cells and localized in stress fibers and cardiocyte Z-disks. J. Biol. Chem. 272, 17542–17550. 10.1074/jbc.272.28.17542 9211900

[B24] Xu Z. Rao Y. Huang Y. Zhou T. Feng R. Xiong S. (2020). Efficient strategies for microglia replacement in the central nervous system. Cell Rep. 32, 108041. 10.1016/j.celrep.2020.108041 32783928

[B25] Yasuhara J. Garg V. (2021). Genetics of congenital heart disease: a narrative review of recent advances and clinical implications. Transl. Pediatr. 10, 2366–2386. 10.21037/tp-21-297 34733677 PMC8506053

[B26] Zhang Q. Gao X. Li C. Feliciano C. Wang D. Zhou D. Mei Y. Monteiro P. Anand M. Itohara S. Dong X. Fu Z. Feng G. (2016). Impaired Dendritic Development and Memory in Sorbs2 Knock-Out Mice. J. Neurosci. 36, 2247–2260. 10.1523/JNEUROSCI.2528-15.2016 26888934 PMC4756157

