# Supplementary material for: Increased cardiac macrophages in Sorbs2-deficient hearts: revealing a potential role for macrophage in responding to embryonic myocardial abnormalities
Source: Front Genet. 2025 Jan 15;15:1525931. doi: 10.3389/fgene.2024.1525931 (PMC11774933; doi:10.3389/fgene.2024.1525931)
Supplement: Supplementary file 5 [file Table6.docx]

***Supplementary Material***

1. **Supplementary Figures**


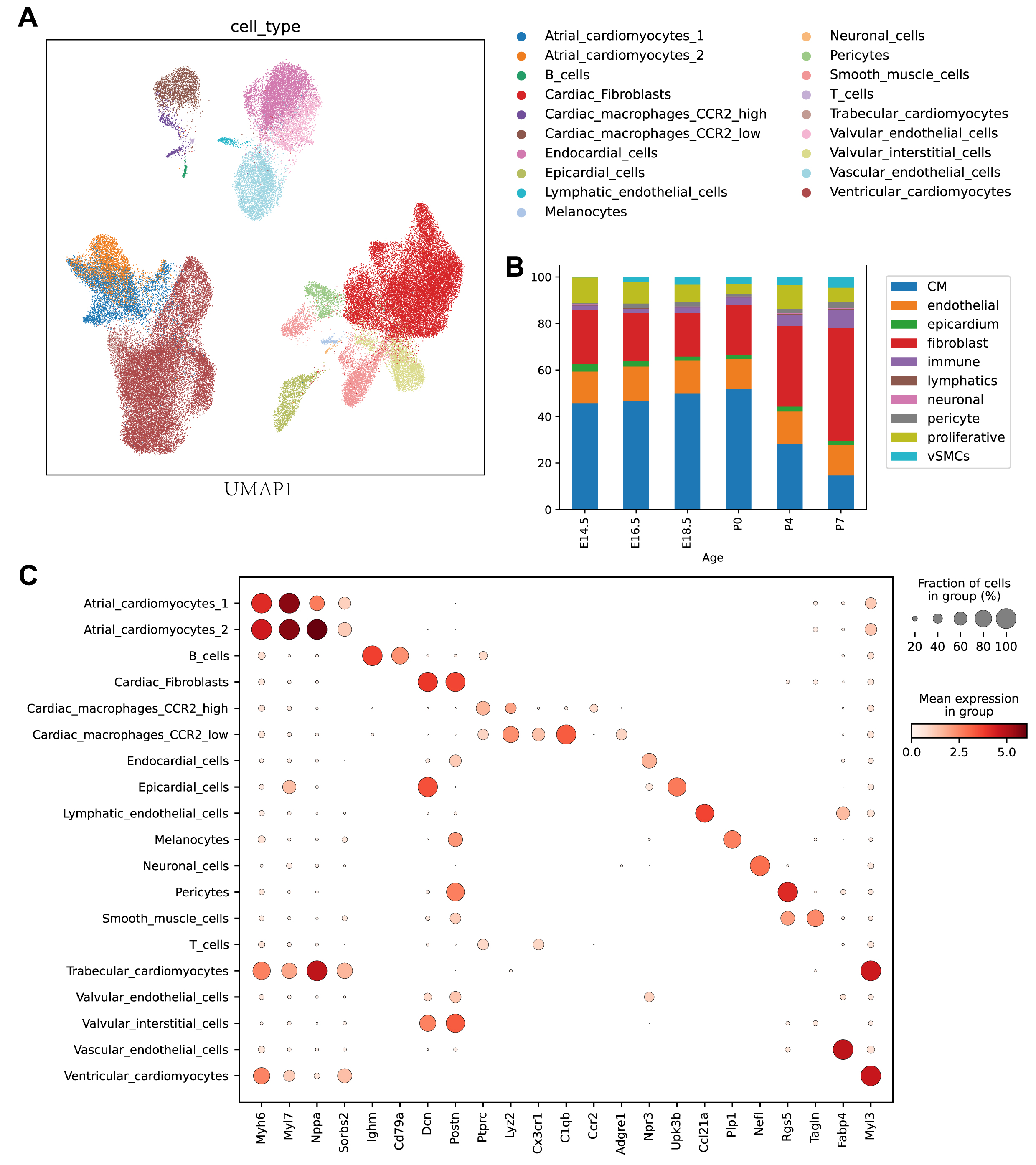


**Supplementary Figure S1. *Sorbs2* is not expressed in macrophages but is highly expressed in cardiomyocytes.**

1. UMAP visualization of single-cell RNA-seq data from the heart across developmental stages E14.5, E16.5, E18.5, P0, P4, and P7. Each dot represents an individual cell, and cells are color-coded according to cell type, highlighting cellular diversity within the heart across these time points. Distinct clusters correspond to different cell types, including cardiomyocytes, endothelial cells, fibroblasts, immune cells, etc.. UMAP dimensionality reduction was applied to visualize relationships between cells based on transcriptomic profiles. Key cell types were annotated using known marker genes.
2. Cell type composition at different developmental stages (E14.5, E16.5, E18.5, P0, P4, P7), shown as percentages. Each bar slice represents the relative proportion of different cell types within the heart. Percentages were calculated based on single-cell RNA-seq data from each stage, indicating developmental shifts in cell type composition.
3. Dotplot showing gene expression profiles across different cell types in the heart at various developmental stages (E14.5, E16.5, E18.5, P0, P4, P7). Each row represents a specific gene, and each column represents a cell type, with dot size corresponding to the percentage of cells expressing the gene and color intensity indicating expression level.
4. **Supplementary Tables**

**Supplementary Table 1. DEGs in E12.5 *Sorbs2^-/-^* hearts (see attached spreadsheet file)**

**Supplementary Table 2. DEGs in E15.5 *Sorbs2^-/-^* hearts (see attached spreadsheet file)**

**Supplementary Table 3. DEGs in E18.5 *Sorbs2^-/-^* hearts (see attached spreadsheet file)**

**Supplementary Table 4. DEGs in macrophage-depleted and non-depleted E12.5 *Sorbs2^-/-^* hearts (see attached spreadsheet file)**

**Supplementary Table 4. DEGs in macrophage-depleted and non-depleted E18.5 *Sorbs2^-/-^* hearts (see attached spreadsheet file)**

**Supplementary Table 6. Genotype distribution in macrophage-depleted and non-depleted E18.5 Embryos.**

| **Embryonic**  **stage** |  | **Control** | | **Macrophage depletion** | |
| --- | --- | --- | --- | --- | --- |
|  | **Total** | **WT or *Sorbs2 ^+/-^*** | ***Sorbs2^-/-^*** | **WT or *Sorbs2^+/-^*** | ***Sorbs2^-/-^*** |
| E18.5 | 180 | 63 | 53 | 36 | 28 |

The observed ratio is not different from the expected. Two-sided χ2 test (χ2=2505, *p*=0.474).
